# Do senior medical students meet recommended emergency medicine curricula requirements?

**DOI:** 10.1186/s12909-017-1110-1

**Published:** 2018-01-05

**Authors:** Sami Shaban, Arif Alper Cevik, Mustafa Emin Canakci, Caglar Kuas, Margret El Zubeir, Fikri Abu-Zidan

**Affiliations:** 1 Department of Medical Education, College of Medicine and Health Sciences, United Arab Emirates University, Al Ain, 17666 United Arab Emirates; 2 Department of Internal Medicine, Emergency Medicine Clerkship, United Arab Emirates University, College of Medicine and Health Sciences, Al Ain, 17666 United Arab Emirates; 3 Department of Emergency Medicine, Eskisehir Osmangazi University, College of Medicine and Health Sciences, Eskisehir, 26350 Turkey; 4 Department of Surgery, College of Medicine and Health Sciences, United Arab Emirates University, Al Ain, 17666 United Arab Emirates

**Keywords:** Emergency medicine, Clerkship, Logbook, Encounter, Curriculum

## Abstract

**Background:**

Emergency departments (EDs) offer a variety of learning opportunities for undergraduate medical students. It is however, difficult to evaluate whether they are receiving recommended training during their emergency medicine (EM) clerkship without identifying their clinical activities. We aimed to evaluate the clinical exposure of the final year medical students at our College during their EM clerkship.

**Methods:**

This is a retrospective analysis of prospectively collected student logbooks. 75 students rotated in a 4-week EM clerkship during 2015–2016. The students rotated in EDs of two hospitals. Each ED treats more than 120,000 cases annually. The students completed 12 eight-hours shifts. Presentations and procedures seen were compared with EM curriculum recommendations.

**Results:**

Five thousand one hundred twenty-two patient presentations and 3246 procedures were recorded in the logbooks, an average (SD) of 68.3 (17.6) patients and 46.1 (14.0) procedures. None of the students encountered all ten recommended presentations. Two students (2.6%) logged all nine procedure categories of the EM curriculum.

**Conclusion:**

Recommended presentations and procedures of the EM clerkship were not fully encountered by all our students. Different settings vary in the availability and type of patients and procedures. Each clinical clerkship should tailor their teaching methods based on the available learning opportunities.

**Electronic supplementary material:**

The online version of this article (10.1186/s12909-017-1110-1) contains supplementary material, which is available to authorized users.

## Background

Emergency Medicine (EM) is an important medical field requiring proper training which is critical for accreditation of medical education programs [1]. Therefore, application of well-established EM curricula in medical schools is vital. EM curriculum development started in the mid-1980s [2, 3]. This curriculum was developed during the last 25 years and implemented in many medical schools. The American College of Emergency Physicians (ACEP), the Society for Academic Emergency Medicine and International Federation for Emergency Medicine are the three main associations which work on the development of medical school curricula [3, 4]. The last curriculum was published in 2010 by the Clerkship Directors Group of Emergency Medicine (CDEM) of Society For Academic Emergency Medicine [5]. Similar presentations and procedures for curriculum were also reported in a different setting by Penciner et al. [6].

Students need a healthy and rich educational environment to improve their skills. Being able to achieve learning objectives is one of the main driving forces for a successful clerkship [7]. Exposure to patients including quantity and severity vary between different settings. Hem-Stokroos et al. showed that individual students’ exposure to various patient problems during a 10-week surgical clerkship was insufficient. The students were not exposed to sufficient quantities of emergency patients [8]. Emergency departments offer rich learning opportunities for undergraduate students because of the variety of acute presentations, significant number of patients, and different emergency procedures. Although a list of clinical presentations has been suggested by the current curriculum [5], a recent study concluded that most students do not encounter all recommended ten emergency presentations [9]. Without identifying the clinical activities that students are experiencing, it is difficult to evaluate whether they are receiving proper training during their EM clerkship [10]. Do our senior medical students meet the recommended curriculum requirements during their rotation? Do they encounter substantial numbers of core presentations and procedures? Answers to these questions are unknown in many EM clerkships. Application of a recommended curriculum in different settings helps to evaluate its applicability and generalizability. Finding similarities or gaps in different settings provides feedback for users and developers of the curricula. We aimed in this study to evaluate the clinical exposure of final year medical students at our college during their EM clerkship.

## Methods

### Ethical approval

This study was reviewed and approved by The Research and Graduate Studies Ethics Committee of United Arab Emirates University (UAEU) (Reference No: ERS-2016-4387).

### Study design and setting

This is a retrospective analysis of prospectively collected data of student logbooks who rotated in a 4-week EM clerkship during 2015–2016 in the College of Medicine and Health Sciences of the UAEU. The EM clerkship is designed based on the curriculum recommendations of the Society for Academic Emergency Medicine and International Federation for Emergency Medicine [4, 5].

Teaching, learning and assessment in the Emergency Medicine clerkship:

The Emergency medicine clerkship includes the following teaching, learning and assessment activities during the 4-week rotation.

- Didactic lectures and case discussions covering the recommended list of core presentations (Additional file 1).
- Skills practice sessions for CPR/arrhythmia management, suturing, airway management, EFAST and RUSH protocols.
- Twelve clinical shifts.
- Assessments include; weekly MCQ exams from the recommended list of core presentations, mini-clinical exams in the clinical shifts, oral case presentations, clinical shift performance evaluations by supervisors, final MCQ exam with 70 questions, and seven OSCE stations.

The EM clerkship students rotate in two teaching hospitals in our city, Tawam-John Hopkins Hospital and Al Ain Hospital. Tawam-John Hopkins Hospital, which is affiliated with Al Ain Medicine International, treats around 127,000 emergency patients in the ED every year. ACGME-I accredited Emergency Medicine Residency Program is also located at this hospital. Al Ain Hospital ED treats around 164,000 emergency patients every year. The students have a total of 12 clinical shifts in these two hospitals during their clerkship and each clinical shift is 8 h long. Clinical shifts are located at four different locations of the ED (resuscitation room, urgent care area, fast track area, and a pediatric unit). The number of shifts were three for resuscitation room, four for urgent care area, two for fast track area, and one for the pediatric unit.

### Participants

Seventy-five final year medical students (49 females, 25 males) were divided into five groups and trained in the EM clerkship. The clerkship runs five times a year with groups of 13–17 students in each rotation.

### Data collection

Our EM clerkship curriculum requires that students examine a minimum of 45 patients (11 recommended presentation categories) and perform 50 procedures (15 recommended procedure categories) by the end of the clerkship (Additional files 1 and 2). Due to the lack of international guideines, the minimum expected numbers, additional topics and procedures were decided by the Clerkship Director and Residency Program Director by the guidance of an EM core faculty questionnaire. The students were guided by the Clerkship Director on how to fill in the logbook during a one-hour orientation session at the beginning of the clerkship. This orientation included the explanation of chief complaints/core presentations and procedures recommended by the curriculum, how students should categorise and log each entry, and deciding the priority of logging the presentation in patients who have multiple chief complaints. They were also informed that they could get help from their supervisor on the clinical shift.. Students were guided to fill the chief complaint area with the patients’ first/chief presenting symptom. Students were also guided to log the most critical/life or organ saving procedures first into their logbooks. However, they were given freedom to log all procedures they did on patients. Data in the logbooks included descriptive information such as patient age and gender, presentation seen and procedure performed by the student, and the student’s involvement level. Student’s involvement had three levels: 1) observation alone (observation with minimal activity), 2) partial involvement (first assistant up to 50% activity) and 3) full involvement (start to finish care more than 50% activity) under supervision. Clinical activities of students were supervised by an attending physician or a senior resident during the clinical shifts. Details of each patient and procedure in the logbook were then reviewed and signed by a supervisor (attending emergency physician or senior EM resident) at the end of the clinical shifts. Supervisors were given the responsibility to accept, modify or cancel the data entry according to their own judgement. All logbooks were collected and evaluated by the EM Clerkship Director at the end of the rotation. The data were manually transferred from the logbooks into an electronic spreadsheet independently by two senior EM residents using two data files. Daily data entry was limited to 2 hto reduce entry errors. The data files of the two residents were compared after the completion of data entry and if there was discrepancy, data were re-examined by the two residents and a consensus reached. If there was no consensus for a specific entry, then the EM Clerkship Director made the final decision.

## Results

Five thousand one hundred twenty-two patient presentations, and 3246 procedures were recorded in the logbooks of all students. An average (SD) of 68.3 (17.6) patients and 46.1 (14.0) procedures. The mean age (SD) of patients was 34.2 (23.4) years. None of the students encountered all recommended presentations by CDEM curriculum (10 presentations). 24 students (32%) encountered between 7 and 9 of CDEM recommended presentations, and 4 students (5.3%) logged 9 presentations.

Abdominal pain, chest pain, and respiratory distress were the leading presentations in the logbook (13.0%, 7.7%, and 5.7% respectively) (Table 1). Altered mental status, shock, and cardiac arrest were the least presentations in logbook (0.7%, 0.4%, and 0.2% respectively). The average number of patients by presentation category and achievement of the students are shown in Table 2. Abdominal pain, chest pain and respiratory distress were also highly achieved by students (93.3%, 92.0%, and 77.3% respectively) while cardiac arrest, shock and gastrointestinal bleeding had the lowest achievement rates (8.0%, 8.0%, and 2.6% respectively). Abdominal pain, chest pain, headache, respiratory distress, and trauma were seen by more than 80% of students. Table 3 shows the details of involvement level and presentation categories logged by students. The students were fully involved in 68.9% of the patients, partially involved in 18.3% and observed in 12.8%. The highest full involvement level by chief complaints were cardiac arrest (100%), shock (80.9%) and fever in a child (78.0%). The level of students’ involvement was statistically different in the different clinical presentations (Pearson’s Chi-squared, *p* = 0.017).

**Table 1:** Presentations and their percentage out of the total patients logged

Presentations	Number of Patients Logged (%)
Abdominal pain	666 (13.0%)
Chest pain	395 (7.7%)
Respiratory distress	293 (5.7%)
Trauma (multiple)	255 (5.0%)
Fever in child^a^	241 (4.7%)
Headache	167 (3.3%)
Poisoning	43 (0.8%)
Altered mental status	38 (0.7%)
Gastrointestinal bleeding	36 (0.7%)
Shock	21 (0.4%)
Cardiac arrest	9 (0.2%)
Other chief complaints	2958 (57.8%)

The presentation list was adopted from CDEM curriculum. ^a^This topic was added by local EM core faculty group. The category of other chief complaints includes presentations not included in the list

**Table 2:** Presentations and students’ level of achievement

Presentations	% of Students Achieved Recommended Number (Additional file 1)	% of Students Logging Presentation	Average of Patients Per Student
Abdominal pain	93.3	100	8.88
Chest pain	92.0	100	5.26
Respiratory distress	77.3	93.3	3.91
Fever in child^a^	65.3	72	3.21
Headache	60.0	84	2.23
Trauma (multiple)	58.7	89.3	3.4
Altered mental status	16.0	24	0.51
Poisoning	13.3	41.3	0.57
Gastrointestinal bleeding	8.0	38.7	0.48
Cardiac arrest	8.0	8	0.12
Shock	2.7	25.3	0.28
Other chief complaints	94.7	100	39.44

The presentation list was adopted from CDEM curriculum. ^a^This topic was added by local EM core faculty group. The category of other chief complaints includes presentations not included in the list

**Table 3:** Presentations and students’ level of involvement

	Involvement Level		
Presentations	Full *N*(%)	Partial *N*(%)	Observation *N*(%)
Abdominal pain	482 (72.4)	99 (14.9)	85 (12.7)
Chest pain	264 (66.8)	74 (18.7)	57 (14.4)
Respiratory distress	209 (71.3)	58 (19.8)	26 (8.8)
Fever in child^a^	188 (78.0)	35 (14.5)	18 (7.47)
Trauma (multiple)	160 (62.7)	54 (21.2)	41 (16.1)
Headache	115 (68.9)	34 (20.3)	18 (10.8)
Poisoning	26 (60.4)	11 (25.6)	6 (14.0)
Altered mental status	29 (76.3)	4 (10.5)	5 (13.2)
Gastrointestinal bleeding	28 (77.8)	6 (16.7)	2 (5.5)
Shock	17 (80.9)	1 (4.8)	3 (14.3)
Cardiac arrest	9 (100.0)	0 (0.0)	0 (0.0)
Other chief complaints	2000 (67.6)	561 (19.0)	397 (13.4)

The presentation list was adopted from CDEM curriculum. ^a^This topic was added by local EM core faculty group. The category of other chief complaints includes presentations not included in the list

None of the students encountered all procedure categories recommended by our core faculty group (15 procedure categories). The average (SD) patient numbers for those procedures per student were 37.5 (12.5). Only two of the students (2.6%) logged all nine procedure categories of the CDEM curriculum. 19 students (25.3%) encountered between 7 and 9 of the CDEM procedure categories. The average (SD) patient numbers for CDEM procedures were 26.7 (8.9) per student. Table 4 shows the main procedures performed by students. Intravenous line placement (37.0%), electrocardiography (ECG) application and interpretation (12.9%), and suturing (9%) were the highest procedures performed by students as recorded in their logbooks. Sedation and analgesia (1.2%), cardiopulmonary resuscitation (0.3%), and lumbar puncture (0.2%) were the least procedures performed. Table 5 shows the level of achievement of students for different procedures. Peripheral intravenous line placement (100%), suturing (92%), ECG application and interpretation (84%) were highly achieved by students. Lowest achievements were seen in lumbar puncture (8%), reduction of dislocations/fractures (6.7%) and nasogastric tube placement (6.7%). ECG application and interpretation, extended focused assessment with sonography for trauma (EFAST), peripheral IV line, and suturing were encountered by more than 80% of the students.

**Table 4:** Procedures logged by students and percentage

	Number of Procedures Logged (%)
Peripheral IV line	1198 (36.9)
ECG application and interpretation	420 (12.9)
Suturing	292 (9.0)
EFAST^a^	135 (4.2)
Splinting/Casting	113 (3.5)
ABG sampling^a^	99 (3.1)
Urinary/foley catheterization	73 (2.2)
NG tube placement	56 (1.7)
Abscess ID	55 (1.7)
Airway management	52 (1.6)
RUSH^a^	48 (1.5)
Reduction of dislocations	46 (1.4)
Sedation and analgesia^a^	38 (1.2)
CPR/Arrythmia management	11 (0.3)
Lumbar puncture^a^	8 (0.2)
Other procedures	602 (18.5)

*ABG* arterial blood gas, *ID* incision and drainage, *CPR* cardiopulmonary resuscitation, *ECG* electrocardiogram, *EFAST* Extended focused assessment with sonography for trauma, *IV* intravenous, *NG* nasogastric, *RUSH* rapid ultrasound for shock and hypotension

The procedure list was adopted from CDEM curriculum. ^a^These procedures were added by local EM core faculty group. The category of other procedures includes procedures not included in the list

**Table 5:** Procedures and student level of achievement

	Number (%) of Students Achieved Recommended Number (Additional file 2)	% of Students Logging Procedure	Average of Procedures per Student
Peripheral IV line	100	100	15.97
Suturing	92.0	98.7	3.89
ECG application and interpretation	84.0	97.3	5.60
Airway management	76.0	76.0	0.69
Abscess ID	52.0	52.0	0.73
Splinting/Casting	48.0	68.0	1.51
EFAST^a^	41.3	82.7	1.80
RUSH^a^	26.7	64.0	0.64
CPR/Arrythmia management	20.0	20.0	0.15
ABG sampling^a^	18.6	61.3	1.32
Sedation and analgesia^a^	13.3	32.0	0.51
Urinary/foley catheterization	8.0	56.0	0.97
Lumbar puncture^a^	8.0	8.0	0.11
Reduction of dislocations	6.7	40.0	0.61
NG tube placement	6.7	37.3	0.75
Other procedures	5.3	98.7	43.28

*ABG* arterial blood gas, *ID* incision and drainage, *CPR* cardiopulmonary resuscitation, *ECG* electrocardiogram, *EFAST* Extended focused assessment with sonography for trauma, *IV* intravenous, *NG* nasogastric, *RUSH* rapid ultrasound for shock and hypotension

The procedure list was adopted from CDEM curriculum. ^a^These procedures were added by local EM core faculty group. The category of other procedures includes procedures not included in the list

Table 6 shows student level of involvement in procedures. Students’ highest full involvement levels were in ECG application and interpretation (72.4%), peripheral intravenous line placement (69.8%) and EFAST (68.1%). Students’ lowest full involvement levels were in airway management applications and procedures (38.5%), lumbar puncture (37.5%), and reduction of fractures or dislocations (37%). The level of students’ involvement was statistically different in the different procedures (Pearson’s Chi-squared, *p* < 0.0001).

**Table 6:** Procedures and Student level of Involvement

	Involvement Level		
	Full *N*(%)	Partial *N*(%)	Observation *N*(%)
Peripheral IV line	836 (69.8)	174 (14.5)	188 (15.7)
ECG application and interpretation^a^	304 (72.4)	74 (17.6)	42 (10.0)
Suturing	158 (54.1)	89 (30.5)	45 (15.4)
EFAST^a^	92 (68.1)	23 (17.0)	20 (14.8)
Splinting/Casting	59 (52.2)	46 (40.7)	8 (7.1)
ABG sampling^a^	50 (50.5)	28 (28.3)	21 (21.2)
Urinary/foley catheterization	45 (61.6)	18 (24.7)	10 (13.7)
Airway management	20 (38.5)	19 (36.5)	13 (25.0)
Reduction of dislocations	17 (37.0)	23 (50.0)	6 (13.0)
Abscess ID	28 (50.9)	24 (43.6)	3 (5.5)
Sedation and analgesia^a^	15 (39.5)	16 (42.1)	7 (18.4)
NG tube placement	34 (60.7)	15 (26.8)	7 (12.5)
RUSH^a^	26 (54.2)	13 (27.1)	9 (18.8)
Lumbar puncture^a^	3 (37.5)	2 (25.0)	3 (37.5)
CPR/Arrythmia management	5 (45.4)	4 (36.4)	2 (18.2)
Other procedures	367 (61.0)	137 (22.8)	98 (16.3)

*ABG* arterial blood gas, *ID* incision and drainage, *CPR* cardiopulmonary resuscitation, *ECG* electrocardiogram, *EFAST* Extended focused assessment with sonography for trauma, *IV* intravenous, *NG* nasogastric, *RUSH* rapid ultrasound for shock and hypotension

The procedure list was adopted from CDEM curriculum. ^a^These procedures were added by local EM core faculty group. The category of other procedures includes procedures not included in the list

## Discussion

Our study has shown that final year medical students encountered less patient presentations and procedures than those recommended by our clerkship curriculum. Abdominal pain, chest pain, and respiratory distress were the highest achieved presentations. Intravenous line placement, ECG application and interpretation, and suturing were the highest achieved procedures.

Emergency departments provide a wide range of presentations and procedures for trainees to encounter, observe and participate in. Clinical activities during shifts are useful educational tools for the EM clerkship [11]. Clinical logbooks are valid to document these activities [12] as they increase student attention to perform medical procedures [13]. It is important to define the learning opportunities so as to improve teaching and learning activities. We can then modify the curriculum depending on each local setting [10].

Avegno et al. reported that about 15% of students examined all recommended presentations of the CDEM curriculum during their EM clerkship [9]. Our findings were similar in common presentations including abdominal and chest pain. Furthermore, their least encounters were shock and cardiac arrest which is similar in our context. Conversely, abdominal and chest pain were seen by 100% of students in the aforementioned study which was also similar to our findings. Cardiac arrest was the only presentation encountered in less than 70% by their students. In our study, this occurred in six presentations. Similar to our findings, McGraw and Lord found that abdominal pain was the most frequent encountered presentation, however, there were no encountered cardiac arrest patients in their study [10].

Suturing was the most commonly performed procedure by students as reported by McGraw and Lord [10], whereas suturing was the third highest encountered procedure in our study. McGraw and Lord showed that students performed an average of one intravenous line insertion [10] compared with 16 in our study. Cardiopulmonary resuscitation was not encountered in their study, and this was also low in our setting. This can be explained by the young age of our population. Nearly 50% of students did not encounter urinary catheterization in McGraw and Lord’s study which is similar to ours [10]. The large number of trainees in Tawam-John Hopkins and Al Ain Hospitals may affect students’ hands-on training. Students may be competing with other residents and trainees in different activities. In addition, some procedures such as urinary catheterization are performed by nurses and their students.

ACEP’s curriculum included 16 knowledge categories such as emergency medical services, cardiovascular diseases, and trauma. There were also specific categories such as ophthalmologic emergencies. There were 15 procedures in the list including basic emergency procedures (gastric lavage, tetanus prophylaxis etc.) as well as cricothyroidotomy and pericardiocentesis. Although emergency departments can provide more exposure than other rotations [14], only about 20% of the ACEP curriculum recommendations could be seen by 80% or more students [15]. In our study, only 5 out of 10 presentations and 2 out of 9 procedure categories, which were recommended by CDEM, were achieved by more than 80% of our students. In the literature, none could achieve 100% completion of all 10 recommended presentations [16, 17]. Nevertheless, setting high standards is useful because it helps us to improve our performance in the clerkship. Furthermore, there are institutional and specialty differences with regard to patient numbers, conditions and achievement level of students [9, 18]. The breadth of clinical experience during an EM clerkship is context specific and dependent on a variety of factors, including case mix and acuity of patient presentations. It is therefore expected to find similarities and differences in the range of presentations and procedures encountered in our study compared with previously published studies.

Medical students show confidence in acute care knowledge, disease management, and procedural skills after completion of an EM clerkship [19]. However, there is no clear description of the level and amount of student involvement during an EM clerkships.

We have added EFAST and rapid ultrasound in shock and hypotension (RUSH) protocols to be achieved by students. Ultrasound training is highly recommended for undergraduate medical education [20]. Our students have been exposed to EFAST and RUSH protocols training since 2013. They are encouraged to use these techniques during clinical shifts. Unsurprisingly, EFAST was encountered in over 80% by of our students while RUSH protocol was encountered by less than 65%.

A strength of this study is its facilitation of recognition of deficient curricular areas needing to be addressed. Our results should nevertheless provide some reassurance to clerkship directors and curriculum developers that the clerkship is providing some very useful opportunities for emergency care encounters. This is particularly important since emergency medicine is being increasingly recognized as an important learning experience for medical students. Furthermore, this conclusion extends across two different EM settings with different patient populations. Low encountered presentations and procedures should be emphasized more in a variety of teaching sessions including simulations [11, 21–23]. Another important finding of our study is that majority of the logged presentations, and 19% of logged procedures were under the ‘other’ category which describes students’ exposure to the presentations and procedures other than the recommended curriculum. Timely feedback to students may guide them in fulfilling the required clerkship objectives. Hard copy logbooks are not useful in giving timely feedback to students. Penciner et al. reported that electronic logging by medical students during an EM clerkship has many advantages [24]. The present study highlights the need for creating an electronic logbook which can regularly check student activities on a daily basis and give continuous feedback.

### Limitations

There was no specific defined number of patients or procedures that are required to be achieved by students in the literature. Terminology in the current EM clerkship guidelines are not specific for logging the presentations [9]. We have to acknowledge that our study has certain limitations. Because of the hardcopy logbook format used in our study, we were not able to analyse more than one complaint of the patients. This underestimated the full exposure of students. Furthermore, supervisors accepted, modified or cancelled the students’ patients or procedures in the logbook according to their judgement. There is a potential categorisation error in this process. However, the supervisors, preceptors and Clerkship Director did their best to assure the validity of our data. Manual data entry may have errors and high-stress shifts may reduce direct supervision of students. It is useful to know whether there is a relationship between the range of presentations encountered by the students and the case mix for each ED. This will provide some insight as to whether a presentation is uncommon for a specific ED or simply a lack of opportunity for students to encounter these presentations despite being relatively common. We had no full data on location, unit, time, and date of encounters. Accordingly we could not analyse this relationship. We have recently developed a new electronic logbook to address this point.

Although students received a one-hour orientation covering how to fill their logbook, students’ decision making for log entries varied, because of variations in symptoms, patients, students, supervisors, and possible multiple complaints in the patients. Our process cannot guarantee that all students act identically when entering presentations. Other authors have indicated that although students should follow the structure and guidance provided by log books this should not be a substitute for a meaningful clinical supervision. Logbooks should be a tool that highlights the importance of quality rather than quantity of patient interactions [25]’.

We also acknowledge that exposure to these patients alone does not assure learning. However, teaching and learning is a holistic body affected by multiple factors. Although we have applied various curriculum delivery modes to achieve the learning outcomes of our students, we cannot completely guarantee that the students met all the desired learning outcomes of the course. There is also a possibility that students enetered data into the logbook depending on their interests or needs which is characteristic of adult learning*.* This might affect the accuracy of reporting. Finally, the study includes a single medical college and 1 year period. Therefore, the generalization of the results may not reflect the reality in a different setting. However, our results were similar to other studies from different parts of the world, which shows limited exposure during the clerkship.

## Conclusion

Recommended presentations and procedures of the EM clerkship were not fully encountered by all of our students. Different settings vary in the available type of patients and procedures. Each clinical clerkship should tailor their teaching methods according to the available clinical learning opportunities. EM Clerkship Directors should monitor their students and their clinical environment targeting to achieve the objectives of their educational curriculum.

## Additional files

Additional file 1: Emergency conditions, presentations and recommended number of patients. (DOCX 11 kb)

Additional file 2: Procedures and recommended numbers. (DOCX 11 kb)

## Abbreviations

ACEP: American College of Emergency Physicians
ACGME-I: Accreditation Council For Graduate Medical Education - International
CDEM: Clerkship Directors Group of Emergency Medicine
ECG: Electrocardiography
EFAST: Extended focused assessment with sonography for trauma
EM: Emergency Medicine
RUSH: Rapid ultrasound for shock and hypotension
UAEU: United Arab Emirates University

## References

[1] American Association of Medical Colleges (AAMC) Liaison Committee on Medical Education (LCME). Functions and structure of a medical school: standards for accreditation of medical education programs leading to the M.D. degree. [cited 2016, June 22]. Available from: http://www.lcme.org/#standard.htm.

[2] Burdick WP, Jouriles NJ, D'Onofrio G (1998). Emergency medicine in undergraduate education. Acad Emerg Med.

[3] Society of Teachers of EmergencyMedicine (STEM) (1985). Core content for undergraduate education in emergency medicine. Ann Emerg Med.

[4] Hobgood C, Anantharaman V, Bandiera G (2009). International Federation for Emergency Medicine model curriculum for medical student education in emergency medicine. Emer Med Australas.

[5] Manthey DE, Ander DS, Gordon DC (2010). Emergency medicine clerkship curriculum: an update and revision. Acad Emerg Med.

[6] Penciner R, Woods RA, McEwen J (2013). Core competencies for emergency medicine clerkships: results of a Canadian consensus initiative. CJEM.

[7] Coates WC (2004). An educator's guide to teaching emergency medicine to medical students. Acad Emerg Med.

[8] Hem-Stokroos HV, Scherpbier AJ, Vleuten CV (2001). How effective is a clerkship as a learning environment?. Medical Teacher.

[9] Avegno J, Leuthauser A, Martinez J (2014). Medical student education in emergency medicine: do students meet the national standards for clinical encounters of selected core conditions?. J Emerg Med..

[10] McGraw R, Lord JA (1997). Clinical activities during a clerkship rotation in emergency medicine. J Emerg Med..

[11] Yeung M, Beecker J, Marks M (2010). A new emergency medicine clerkship program: students' perceptions of what works. CJEM.

[12] Soler NG, Mast TA, Anderson MB, Kienzler LMA (1981). Logbook system for monitoring student skills and experiences. J Med Educ.

[13] Hunskaar S, Seim SH (1984). The effect of a checklist on medical students’ exposures to practical skills. Med Educ.

[14] Johnson GA, Pipas L, Newman-Palmer NB, Brown LH (2002). The emergency medicine rotation: a unique experience for medical students. J Emerg Med.

[15] De Lorenzo RA, Mayer D, Geehr EC (1990). Analyzing clinical case distributions to improve an emergency medicine clerkship. Ann Emerg Med.

[16] Lampe CJ, Coates WC, Gill AM (2008). Emergency medicine subintern- ship: does a standard clinical experience improve performance out- comes?. Acad Emerg Med.

[17] Coates WC, Gendy MS, Gill AM (2003). Emergency medicine subintern- ship: can we provide a standard clinical experience?. Acad Emerg Med.

[18] Ferenchick G, Mohmand A, Mireles J, Solomon D (2009). Using patient encounter logs for mandated clinical encounters in an internal med- icine clerkship. Teach Learn Med.

[19] Avegno JL, Murphy-Lavoie H, Lofaso DP, Moreno-Walton L (2012). Medical students’ perceptions of an emergency medicine clerkship: an analysis of self-assessment surveys. Int J Emerg Med.

[20] Favot M, Courage C, Mantouffel J, Amponsah D (2015). Ultrasound training in the emergency medicine clerkship. West J Emerg Med.

[21] Takayesu JK, Farrell SE, Evans AJ (2006). How do clinical clerkship students experience simulator-based teaching? A qualitative analysis. Simul Healthc.

[22] Goolsby CA, Goodwin TL, Vest RM (2014). Hybrid simulation improves medical student procedural confidence during EM clerkship. Mil Med.

[23] Sricharoen P, Yuksen C, Sittichanbuncha Y, Sawanyawisuth K (2015). Teaching emergency medicine with workshops improved medical student satisfaction in emergency medicine education. Adv Med Educ Pract.

[24] Penciner R, Siddiqui S, Lee S (2007). Emergency medicine clerkship encounter and procedure logging using handheld computers. Acad Emerg Med.

[25] Gouda P (2016). The need for logbooks to evolve in the undergraduate medical setting. Perspectives on Medical Education.

