# Supplementary material for: Do senior medical students meet recommended emergency medicine curricula requirements?
Source: BMC Med Educ. 2018 Jan 5;18:8. doi: 10.1186/s12909-017-1110-1 (PMC5756377; doi:10.1186/s12909-017-1110-1)
Supplement: Supplementary file 1 — Emergency conditions, presentations and recommended number of patients. (DOCX 11 kb) [file 12909_2017_1110_MOESM1_ESM.docx]

**Additional file 1:** Emergency conditions, presentations and recommended number of patients

| **Presentations** | **Recommended Number** |
| --- | --- |
| Abdominal pain | 3 |
| Altered mental status | 2 |
| Cardiac arrest | 1 |
| Chest pain | 3 |
| Fever in child* | 2 |
| Gastrointestinal bleeding | 2 |
| Headache | 2 |
| Poisoning | 2 |
| Respiratory distress | 2 |
| Shock | 2 |
| Trauma (multiple) | 3 |
| Other chief complaints | 20 |

ABG: arterial blood gas; ID: incision and drainage, CPR: cardiopulmonary resuscitation; ECG: electrocardiogram; EFAST: Extended focused assessment with sonography for trauma; IV: intravenous; NG: nasogastric; RUSH: rapid ultrasound for shock and hypotension.

The presentation list was adopted from CDEM curriculum. * This topic was added by local EM core faculty group. The category of other chief complaints includes presentations not included in the list.
