# Supplementary material for: Do senior medical students meet recommended emergency medicine curricula requirements?
Source: BMC Med Educ. 2018 Jan 5;18:8. doi: 10.1186/s12909-017-1110-1 (PMC5756377; doi:10.1186/s12909-017-1110-1)
Supplement: Supplementary file 2 — Procedures and recommended numbers. (DOCX 11 kb) [file 12909_2017_1110_MOESM2_ESM.docx]

**Additional file 2:** Procedures and recommended numbers

| **Presentations** | **Recommended Number** | |  |
| --- | --- | --- | --- |
| ABG sampling* | | 3 | |
| Abscess ID | | 1 | |
| Airway management | | 1 | |
| CPR/Arrythmia management | | 1 | |
| ECG application and interpretation | | 3 | |
| EFAST* | | 3 | |
| Peripheral IV line | | 3 | |
| Lumbar puncture* | | 1 | |
| NG tube placement | | 3 | |
| Reduction of dislocations | | 2 | |
| RUSH* | | 2 | |
| Sedation and analgesia* | | 2 | |
| Splinting/Casting | | 2 | |
| Suturing | | 2 | |
| Urinary/foley catheterization | | 3 | |
| Other procedures | | 18 | |

ABG: arterial blood gas; ID: incision and drainage, CPR: cardiopulmonary resuscitation; ECG: electrocardiogram; EFAST: Extended focused assessment with sonography for trauma; IV: intravenous; NG: nasogastric; RUSH: rapid ultrasound for shock and hypotension.

The procedure list was adopted from CDEM curriculum. * These procedures were added by local EM core faculty group. The category of other procedures includes procedures not included in the list.
